# Unraveling the magnetic softness in Fe–Ni–B-based nanocrystalline material by magnetic small-angle neutron scattering

**DOI:** 10.1107/S2052252521010605

**Published:** 2021-11-19

**Authors:** Mathias Bersweiler, Michael P. Adams, Inma Peral, Joachim Kohlbrecher, Kiyonori Suzuki, Andreas Michels

**Affiliations:** a Department of Physics and Materials Science, Université du Luxembourg, 162A avenue de la Faïencerie, L-1511 Luxembourg, Grand Duchy of Luxembourg; b Laboratory for Neutron Scattering, ETH Zurich and Paul Scherrer Institut, Villigen PSI 5232, Switzerland; c Department of Materials Science and Engineering, Monash University, Clayton, Victoria 3800, Australia

**Keywords:** small-angle neutron scattering, micromagnetic theory, soft magnetic materials, nanocrystalline alloys, materials science, magnetic scattering, magnetic structures, inorganic materials, nanostructures

## Abstract

Magnetic small-angle neutron scattering is employed to investigate the magnetic interactions in (Fe_0.7_Ni_0.3_)_86_B_14_ alloy, a HiB-NANOPERM-type soft magnetic nanocrystalline material, which exhibits an ultrafine microstructure with an average grain size below 10 nm. The neutron data reveal a significant spin-misalignment scattering which is mainly related to the jump of the longitudinal magnetization at internal particle–matrix interfaces. The field dependence of the neutron data can be well described by micromagnetic small-angle neutron scattering theory. In particular, the theory explains the ‘clover-leaf-type’ angular anisotropy observed in the purely magnetic neutron scattering cross section. The presented neutron data analysis also provides access to the magnetic interaction parameters, such as the exchange-stiffness constant, which plays a crucial role towards the optimization of the magnetic softness of Fe-based nanocrystalline materials.

## Abstract 2

Magnetic field-dependent small-angle neutron scattering is employed to analyze the mesoscale magnetic interactions in a soft magnetic HiB-NANOPERM-type alloy and relate the parameters to the experimental coercivity.

## 1. Introduction

Since the pioneering work of Yoshizawa *et al.* (1988 ▸), the development of novel Fe-based nanocrystalline soft magnetic materials raised considerable interest owing to their great potential for technological applications (Petzold, 2002 ▸; Makino *et al.*, 1997 ▸). The most well known examples are FINEMET- (Yoshizawa *et al.*, 1988 ▸), VITROPERM-(Vacuumschmelze GmbH, 1993 ▸) and NANOPERM-type (Suzuki *et al.*, 1991 ▸) soft magnetic alloys, which find widespread application as magnetic cores in high-frequency power transformers or in interface transformers in the ISDN-telecommunication network. For a brief review of the advances in Fe-based nanocrystalline soft magnetic alloys, we refer the reader to the article by Suzuki *et al.* (2019 ▸).

More recently, an ultra-fine-grained microstructure combined with excellent soft magnetic properties was obtained in HiB-NANOPERM-type alloys (Li *et al.*, 2020 ▸). The magnetic softness in such materials can be attributed to the exchange-averaging effect of the local magnetocrystalline anisotropy *K*_1_. This phenomenon has been successfully modeled within the framework of the random anisotropy model (RAM) (Herzer, 1989 ▸, 1990 ▸, 2007 ▸; Suzuki *et al.*, 1998 ▸), and becomes effective when the average grain size *D* is smaller than the ferromagnetic exchange length 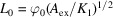 , where *A*_ex_ is the exchange-stiffness constant and φ_0_ is a proportionality factor of the order of unity which reflects the symmetry of *K*_1_. In this regime, the RAM predicts that the coercivity *H*_C_ scales as 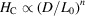 , where *n* = 3 or *n* = 6 depending on the nature of the magnetic anisotropy [see, for example, the work by Suzuki *et al.* (1998 ▸, 2019 ▸) for details]. Therefore, an improvement of the magnetic softness comes about by either reducing *D* and/or increasing *L*_0_.

In the context of increasing *L*_0_, the quantitative knowledge of *A*_ex_ could help to further develop novel Fe-based soft magnetic nanocrystalline materials. However, up to now, most of the research activities in this field are focused on the overall characterization, *e.g.* via hysteresis-loop measurements (coercivity, saturation magnetization and permeability) and magnetic anisotropy determination (crystalline, shape or stress related) (McHenry *et al.*, 1999 ▸; Herzer, 2013 ▸; Suzuki *et al.*, 2019 ▸). One reason for this might be related to the fact that many of the conventional methods for measuring *A*_ex_ (*e.g.* magneto-optical, Brillouin light scattering, spin-wave resonance or inelastic neutron scattering) require thin-film or single-crystal samples.

In the present work, we employ magnetic field-dependent small-angle neutron scattering (SANS) to determine the magnetic interaction parameters in (Fe_0.7_Ni_0.3_)_86_B_14_ alloy, specifically, the exchange-stiffness constant and the strength and spatial structure of the magnetic anisotropy and magnetostatic fields. The particular alloy under study is a promising HiB-NANOPERM-type soft magnetic material, which exhibits an ultra-fine microstructure with an average grain size below 10 nm (Li *et al.*, 2020 ▸). Magnetic SANS is a unique and powerful technique to investigate the magnetism of materials on the mesoscopic length scale of ∼1–300 nm [*e.g.* nanorod arrays (Grigoryeva *et al.*, 2007 ▸; Günther *et al.*, 2014 ▸; Maurer *et al.*, 2014 ▸), nanoparticles (Bender *et al.*, 2019 ▸, 2020 ▸; Bersweiler *et al.*, 2019 ▸; Zákutná *et al.*, 2020 ▸; Kons *et al.*, 2020 ▸; Köhler *et al.*, 2021 ▸), INVAR alloy (Stewart *et al.*, 2019 ▸) or nanocrystalline materials (Ito *et al.*, 2007 ▸; Mettus & Michels, 2015 ▸; Titov *et al.*, 2019 ▸; Oba *et al.*, 2020 ▸; Bersweiler *et al.*, 2021 ▸)]. For a summary of the fundamentals and the most recent applications of the magnetic SANS technique, we refer the reader to the literature (Mühlbauer *et al.*, 2019 ▸; Michels, 2021 ▸).

This paper is organized as follows: Section 2 provides some details of the sample characterization and the neutron experiment. Section 3 summarizes the main expressions for the magnetic SANS cross section and describes the data-analysis procedure to obtain the exchange constant and the average magnetic anisotropy field and magnetostatic field. Section 4 presents and discusses the experimental results, while Section 5 summarizes the main findings of this study.

## 2. Experimental

The ultra-rapidly annealed (Fe_0.7_Ni_0.3_)_86_B_14_ alloy (HiB-NANOPERM-type) was prepared according to the synthesis process detailed by Li *et al.* (2020 ▸). The sample for the neutron experiment was prepared by employing the low-capturing isotope ^11^B as the starting material. The average crystallite size was estimated by wide-angle X-ray diffraction (XRD) using a Bruker D8 diffractometer in Bragg–Brentano geometry (Cu *K*α radiation source). The magnetic measurements were performed at room temperature using a Cryogenic Ltd vibrating sample magnetometer equipped with a 14 T superconducting magnet and a Riken Denshi BHS-40 DC hysteresis loop tracer. The crystallization and Curie temperatures were determined by means of differential thermal analysis (DTA) and thermo-magneto-gravimetric analysis (TMGA) on Perkin Elmer DTA/TGA 7 analyzers under a constant heating rate of 0.67 K s^−1^. For the neutron experiments, six (Fe_0.7_Ni_0.3_)_86_B_14_ ribbons with a surface area of 12 × 20 mm and a thickness of ∼15 µm were stacked together, resulting in a total sample thickness of ∼90 µm. The neutron measurements were conducted at the instrument SANS-1 at the Swiss Spallation Neutron Source at the Paul Scherrer Institute, Switzerland. We used an unpolarized incident neutron beam with a mean wavelength of λ = 6.0 Å and a wavelength broadening of Δλ/λ = 10% (full width at half-maximum). All neutron measurements were conducted at room temperature and within a *q*-range of about 0.036 nm^−1^ ≤ *q* ≤ 1.16 nm^−1^. A magnetic field **H**_0_ was applied perpendicular to the incident neutron beam (**H**_0_ ⊥ **k**_0_). Neutron data were recorded by decreasing the field from the maximum field available of 8.0 to 0.02 T following the magnetization curve (see Fig. 2). The internal magnetic field *H*_i_ was estimated as 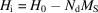 , where *M*_S_ is the saturation magnetization and *N*_d_ is the demagnetizing factor, which was determined based on the analytical expression given for a rectangular prism (Aharoni, 1998 ▸). Neutron data reduction (corrections for background scattering and sample transmission) was conducted using the *GRASP* software package (Dewhurst, 2018 ▸).

## 3. Micromagnetic SANS theory

### 3.1. Unpolarized SANS

Based on the micromagnetic SANS theory for two-phase particle–matrix-type ferromagnets developed by Honecker & Michels (2013 ▸), the elastic total (nuclear + magnetic) unpolarized SANS cross section dΣ/dΩ at momentum-transfer vector **q** can be formally written as (**H**_0_ ⊥ **k**_0_):

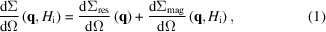

where

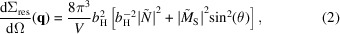

corresponds to the (nuclear + magnetic) residual SANS cross section, which is measured at complete magnetic saturation, and

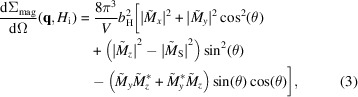

denotes the purely magnetic SANS cross section. In Equations (1)–(3), *V* is the scattering volume; *b*_H_ = 2.91 × 10^8^ Å^−1^ m^−1^ relates the atomic magnetic moment to the atomic magnetic scattering length; 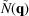 and 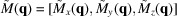 represent the Fourier transforms of the nuclear scattering length density *N*(**r**) and of the magnetization vector field **M**(**r**), respectively; θ specifies the angle between **H**_0_ and **q** ≃ *q*{0, sin(θ), cos(θ)} in the small-angle approximation; and the asterisks (*) denote the complex conjugated quantities. 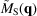 is the Fourier transform of the saturation magnetization profile *M*_S_(**r**), *i.e.* 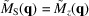 at complete magnetic saturation [compare Equation (2)]. For small-angle scattering, the component of the scattering vector along the incident neutron beam, here *q_x_*, is smaller than the other two components *q_y_* and *q_z_*, so that only correlations in the plane perpendicular to the incoming neutron beam are probed.

In our neutron-data analysis, to experimentally access dΣ_mag_/dΩ, we subtracted the SANS cross section dΣ/dΩ measured at the largest available field (approach-to-saturation regime; compare Fig. 2 ▸) from 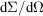 measured at lower fields. This specific subtraction procedure eliminates the nuclear SANS contribution 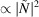 , which is field independent, and therefore

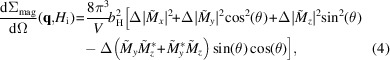

where Δ represents the differences of the Fourier components at the two selected fields (low field minus highest field).

### 3.2. Approach-to-saturation regime

In the particular case of the approach-to-saturation regime, where 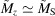 , and which implies therefore 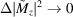 in Equation (4), dΣ/dΩ can be re-written as:

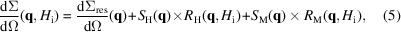

where 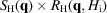 and 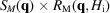 correspond to the magnetic scattering contributions due to perturbing magnetic anisotropy fields and magnetostatic fields, respectively. More specifically, the anisotropy-field scattering function

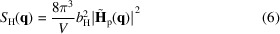

depends of the Fourier coefficient 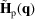 of the magnetic anisotropy field, whereas the scattering function of the longitudinal magnetization

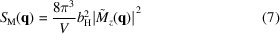

is related to the Fourier coefficient 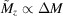 . For an inhomogeneous material of the NANOPERM-type, the latter quantity is related to the magnetization jump Δ*M* at internal (*e.g.* particle–matrix) interfaces. We would like to emphasize that the **q** dependence of *S*_H_ and *S*_M_ can often be described by a particle form factor (*e.g.* sphere) or a Lorentzian-squared function. The corresponding (dimensionless) micromagnetic response functions *R*_H_ and *R*_M_ are given by

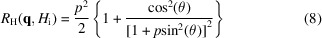

and

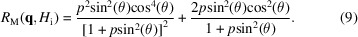

The dimensionless function 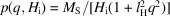 depends on the internal magnetic field *H*_i_ and on the exchange length 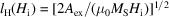 .

### 3.3. Estimation of the magnetic interaction parameters

Most of the time it is more convenient to analyze the (over 2π) azimuthally averaged SANS cross sections instead of the 2D ones. By performing an azimuthal average of the response functions [Equations (8) and (9)] with respect to the angle θ, *i.e.* 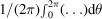 , and by assuming *S*_H_ and *S*_M_ to be isotropic (θ-independent), the SANS cross section dΣ/dΩ can be written as:

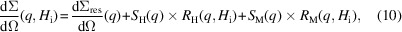

where

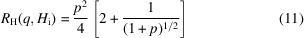

and

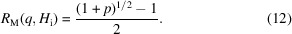

For a given set of parameters *A*_ex_ and *M*_S_, the numerical values of *R*_H_ and *R*_M_ are known at each value of *q* and *H*_i_. Because of the linearity of Equation (10) in *R*_H_ and *R*_M_, one can obtain the values of 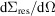 (as the intercept) and *S*_H_ and *S*_M_ (as the slopes) at each *q*-value by performing a (weighted) non-negative least-squares fit of the azimuthally averaged SANS cross sections dΣ/dΩ measured at several *H*_i_. Treating *A*_ex_ in the expression for 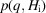 as an adjustable parameter during the fitting procedure allows us to estimate this quantity. The best-fit value for *A*_ex_ is obtained from the minimization of the (weighted) mean-squared deviation between experiment and fit:

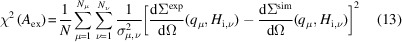

where the indices μ and ν refer to the particular *q* and *H*_i_-values, 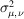 denotes the uncertainties in the experimental data, *N* = *N*_μ_ *N*_ν_ corresponds to the number of data points, and dΣ^exp^/dΩ and dΣ^sim^/dΩ are the azimuthally averaged SANS cross section determined from the neutron experiments and numerically computed using Equation (10), respectively. We would like to point out that the best-fit value for *A*_ex_ represents an average over the sample volume.

Finally, the numerical integration of the determined *S*_H_(*q*) and *S*_M_(*q*) over the whole-**q** space according to the work by Honecker & Michels (2013 ▸)

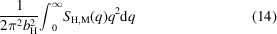

yields the mean-square anisotropy field 〈∣**H**_p_∣^2^〉 and the mean-square longitudinal magnetization fluctuation 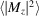 , respectively. Since the neutron experiments are performed within a finite *q*-range and since both integrands 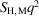 do not exhibit any sign of convergence, one can only obtain a lower bound for both quantities by numerical integration. Moreover, it is important to realize that the specific neutron data analysis described above does not represent a ‘continuous’ fit of dΣ/dΩ in the conventional sense, but rather the point-by-point reconstruction of the theoretical cross sections based on the experimental data.

## 4. Results and discussion

**Figure 1:**
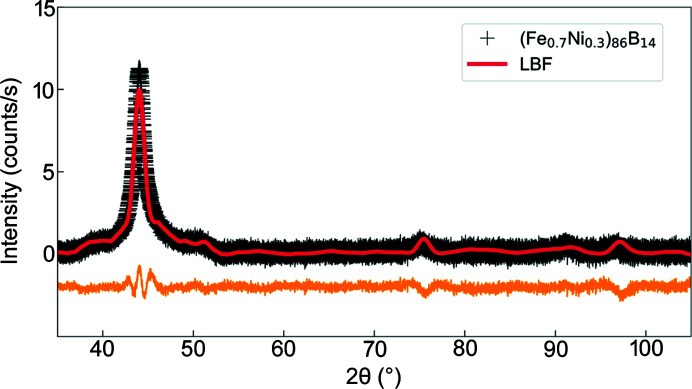
XRD pattern for (Fe_0.7_Ni_0.3_)_86_B_14_ ribbons, a HiB-NANOPERM-type soft magnetic nanocrystalline material (black crosses; Cu *K*α radiation). Red solid line: XRD data refinement using the LBF method implemented in the *FullProf* software. The bottom orange solid line represents the difference between the calculated and experimental intensities.

Fig. 1 ▸ displays the wide-angle XRD results of the (Fe_0.7_Ni_0.3_)_86_B_14_ ribbons. The XRD pattern exhibits only the reflections from the f.c.c.-Fe(Ni) phase, as expected for this particular composition (Li *et al.*, 2020 ▸), and therefore confirms the high-quality synthesis of the sample. The values of the lattice parameter *a* and the average crystallite size *D* were estimated from the XRD data refinement using the LeBail fit method (LBF) implemented in the *FullProf* suite (Rodríguez-Carvajal, 1993 ▸). The best-fit values are summarized in Table 1 ▸. Both values are consistent with the data in the literature [compare the work by Anand *et al.* (2019 ▸) and Li *et al.* (2020 ▸) for *a* and *D*, respectively]. As previously discussed, the origin of the exceptionally fine microstructure observed in (Fe_0.7_Ni_0.3_)_86_B_14_ alloys may be qualitatively attributed to the ultrafast nucleation kinetics of the f.c.c.-Fe(Ni) phase (Li *et al.*, 2020 ▸).

**Figure 2:**
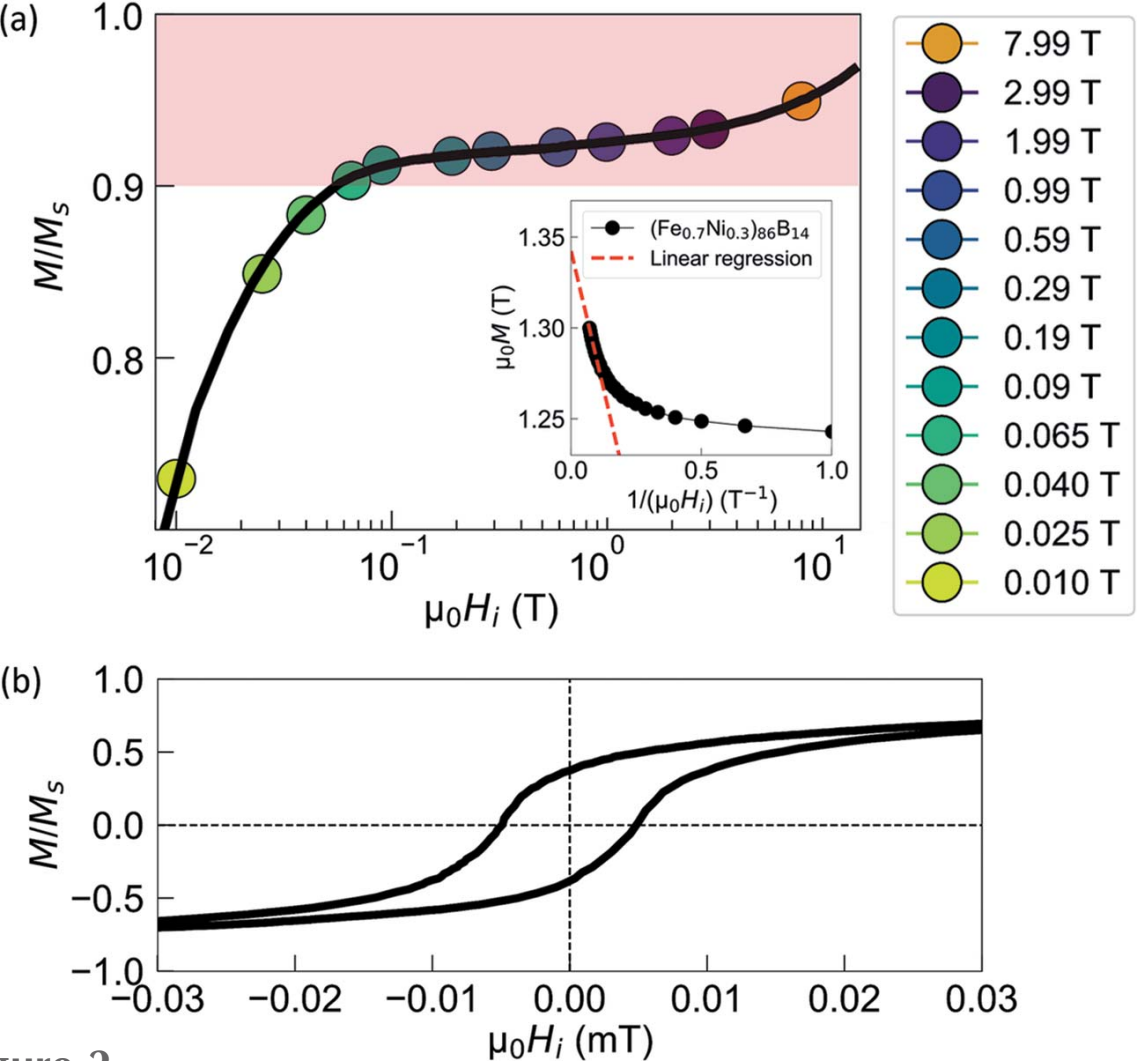
(*a*) Normalized positive magnetization branch measured at room temperature (semi-logarithmic scale). Color-filled circles: *M*/*M*_S_ values for which the SANS measurements have been performed. The approach-to-saturation regime, defined as *M*/*M*_S_ ≥ 90%, is indicated by the red-shaded area. Inset: plot of the magnetization as a function of 1/*H*_i_ (black circles). Red dashed line: linear regression for 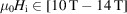 (linear–linear scale). (*b*) Normalized magnetization curve measured using a Riken Denshi BHS-40 DC hysteresis loop tracer, revealing a coercivity of μ_0_ *H*_C_ ≃ 0.0049 mT (linear–linear scale).

Fig. 2 ▸(*a*) presents the positive magnetization branch on a semi-logarithmic scale (measured at room temperature), while the hysteresis loop on a linear–linear scale, and between ±0.03 mT, is displayed in Fig. 2 ▸(*b*). The data have been normalized by the saturation magnetization *M*_S_, which was estimated from the linear regression 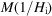 for 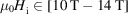 [see inset in Fig. 2 ▸(*a*)]. The values of *M*_S_ and *H*_C_ (see Table 1 ▸) are in agreement with those reported in the literature (Li *et al.*, 2020 ▸). Defining the approach-to-saturation regime by *M*/*M*_S_ ≥ 90%, we can see that this regime is reached for μ_0_ *H*_i_ ≳ 65 mT. Moreover, the extremely small value for *H*_C_ combined with the high *M*_S_ confirms the huge potential of (Fe_0.7_Ni_0.3_)_86_B_14_ alloy as a soft magnetic material, and suggests that in the framework of the RAM (Herzer, 2007 ▸), *H*_C_ should fall into the regime where 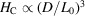 (Suzuki *et al.*, 2019 ▸).

**Figure 3:**
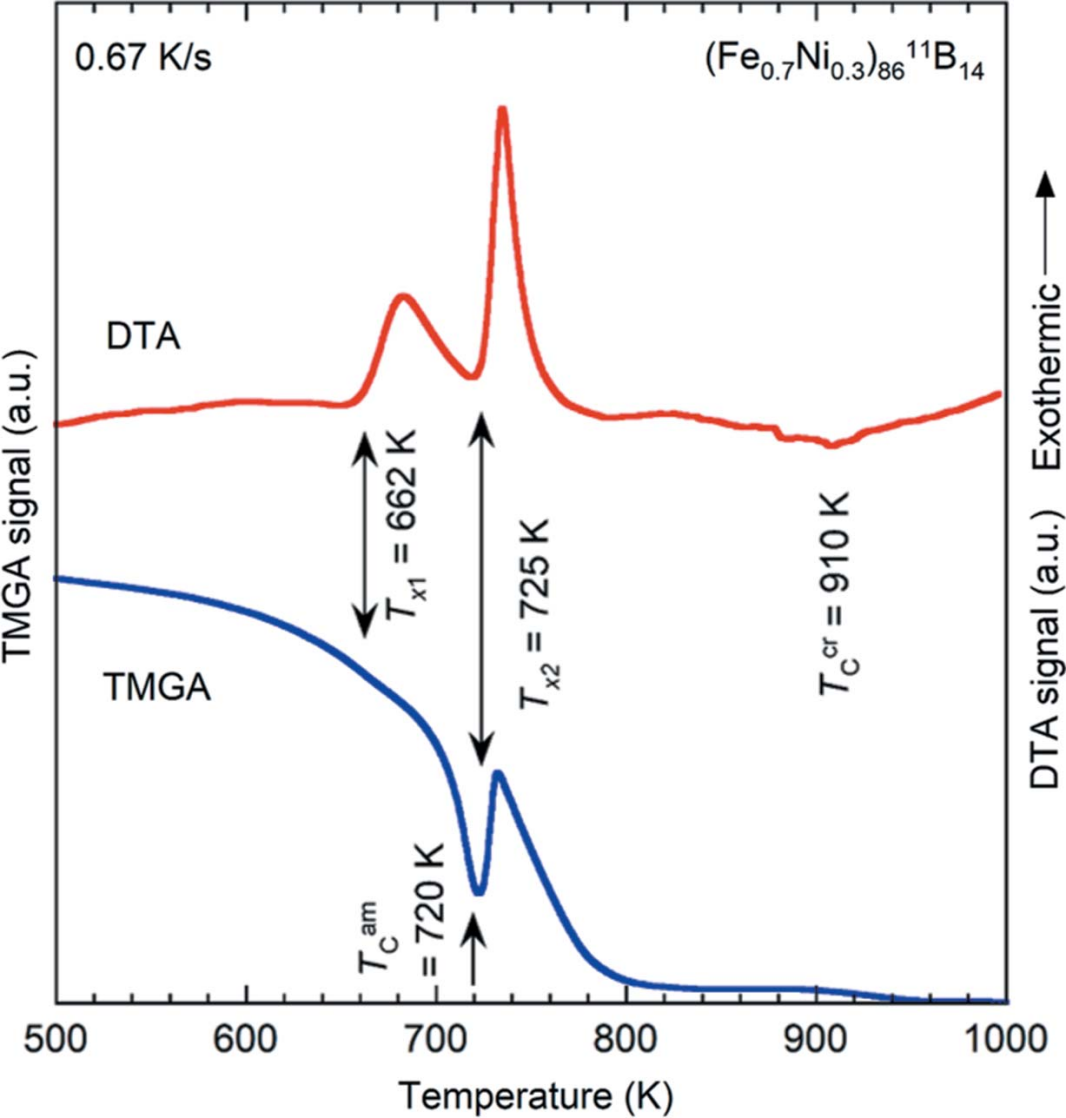
Results of DTA (red solid line) and TMGA (blue solid line) for amorphous (Fe_0.7_Ni_0.3_)_86_B_14_ alloy. The arrows mark the crystallization and Curie temperatures.

Fig. 3 ▸ shows the DTA and TMGA curves for the amorphous (Fe_0.7_Ni_0.3_)_86_B_14_ alloy. Two exothermic peaks are evident on the DTA curve reflecting the well known two-stage reactions, where f.c.c.-Fe(Ni) forms at the first peak followed by decomposition of the residual amorphous phase at the second peak. The sharp drop of the TMGA signal just before the second stage crystallization corresponds to the Curie temperature of the residual amorphous phase ( 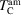 ≃ 720 K). This value, which reflects the exchange integral in our sample (see below), is consistent with those determined for amorphous Fe_86_B_14_ samples prepared under similar conditions (Zang *et al.*, 2020 ▸).

**Figure 4:**
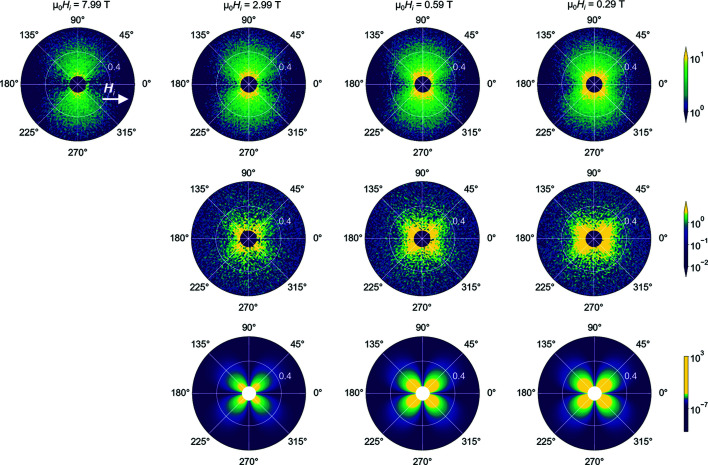
Experimental 2D total (nuclear + magnetic) SANS cross section dΣ/dΩ of (Fe_0.7_Ni_0.3_)_86_B_14_ alloy at the selected fields 7.99, 2.99, 0.59, 0.29 T (upper row), and the corresponding purely magnetic SANS cross section dΣ_mag_/dΩ (middle row). Experimental dΣ_mag_/dΩ were obtained by subtracting dΣ/dΩ at the (near-) saturation field of 7.99 T from the data at the lower fields. The applied (internal) magnetic field *H*_i_ is horizontal in the plane of the detector 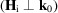 . Lower row: computed dΣ_mag_/dΩ based on the micromagnetic SANS theory [Equations (5)–(9)] at the same selected field values as above, and using the experimental parameters given in Table 1 ▸. Note that dΣ/dΩ and dΣ_mag_/dΩ are plotted in polar coordinates with *q* (nm^−1^), θ (°) and intensity (cm^−1^).

Fig. 4 ▸ (upper row) shows the experimental 2D total (nuclear + magnetic) SANS cross sections dΣ/dΩ of the (Fe_0.7_Ni_0.3_)_86_B_14_ ribbons at different selected fields. As can be seen, at μ_0_ *H*_i_ = 7.99 T (near saturation), the pattern is predominantly elongated perpendicular to the magnetic field direction. This particular feature in dΣ/dΩ is the signature of the so-called ‘ 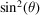 -type’ angular anisotropy [compare Equation (2)]. Near saturation, the magnetic scattering resulting from the spin misalignment is small compared with that resulting from the longitudinal magnetization jump at the internal (*e.g.* particle–matrix) interfaces. By reducing the field, the patterns remain predominantly elongated perpendicular to the magnetic field, but at the smaller momentum transfers *q* an additional field-dependent signal is observed ‘roughly’ along the diagonals of the detector, suggesting a more complex magnetization structure. Fig. 4 ▸ (middle row) presents the corresponding 2D purely magnetic SANS cross sections dΣ_mag_/dΩ determined by subtracting dΣ/dΩ at μ_0_ *H*_i_ = 7.99 T from the data at lower fields. In this way, the maxima along the diagonals of the detector become more clearly visible, thereby revealing the so-called ‘clover-leaf-type’ angular anisotropy pattern. This particular feature was also previously observed in NANOPERM-type soft magnetic merials (Honecker *et al.*, 2013 ▸), and is related to the dominant magnetostatic term *S*_M_ × *R*_M_ in the expression for dΣ_mag_/dΩ [compare Equations (8) and (9)]. More specifically, the jump in the magnitude of the saturation magnetization at the particle–matrix interfaces, which can be of the order of 1 T in these type of alloys (Honecker *et al.*, 2013 ▸), results in dipolar stray fields which produce spin disorder in the surroundings. Fig. 4 ▸ (lower row) displays dΣ_mag_/dΩ computed using the micromagnetic SANS theory [Equations (5)–(9)] and the experimental parameters summarized in Table 1 ▸. As is seen, the clover-leaf-type angular anisotropy experimentally observed in Fig. 4 ▸ (middle row) can be well reproduced using micromagnetic theory.

**Figure 5:**
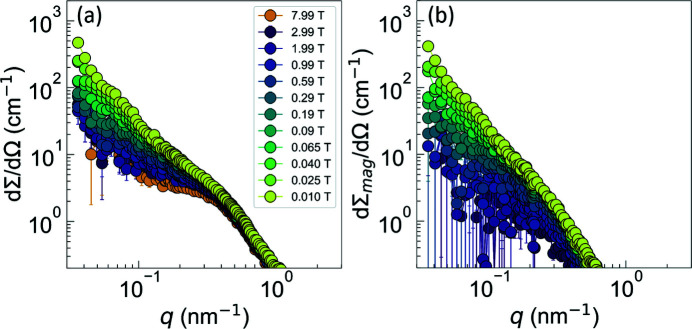
(*a*) Magnetic field dependence of the (over 2π) azimuthally averaged total (nuclear + magnetic) SANS cross section dΣ/dΩ of (Fe_0.7_Ni_0.3_)_86_B_14_ alloy. (*b*) The corresponding purely magnetic SANS cross section dΣ_mag_/dΩ (log–log scale).

Fig. 5 ▸(*a*) displays the (over 2π) azimuthally averaged dΣ/dΩ, while the corresponding dΣ_mag_/dΩ are shown in Fig. 5 ▸(*b*). By decreasing μ_0_ *H*_i_ from 7.99 T to 10 mT, the intensity of dΣ/dΩ increases by almost two orders of magnitude at the smallest momentum transfers *q*. By comparison to Equations (1)–(4), it appears obvious that the magnetic field dependence of dΣ/dΩ can only result from the mesoscale spin disorder (*i.e.* from the failure of the spins to be fully aligned along **H**_0_). As is seen in Fig. 5 ▸(*b*), the magnitude of dΣ_mag_/dΩ is of the same order as dΣ/dΩ, supporting the notion of dominant spin-misalignment scattering in (Fe_0.7_Ni_0.3_)_86_B_14_ alloy.

**Figure 6:**
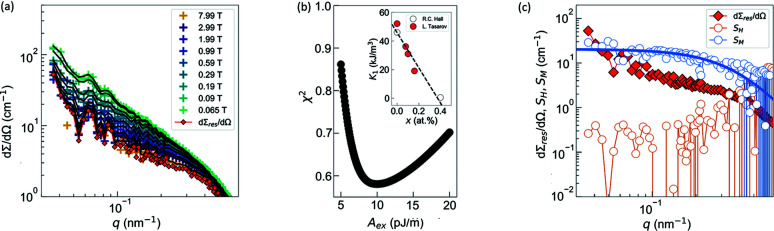
Results of the SANS data analysis of (Fe_0.7_Ni_0.3_)_86_B_14_ alloy. (*a*) Magnetic field dependence of the (over 2π) azimuthally averaged total (nuclear + magnetic) SANS cross section dΣ/*d*Ω plotted in Fig. 5 ▸(*a*) along with the corresponding fits (black solid lines) based on the micromagnetic SANS theory [Equation (10)]. (*b*) Weighted mean-squared deviation between experiment and fit, χ^2^, determined using Equation (13) as a function of the exchange-stiffness constant *A*_ex_. Inset: Fe-composition dependence of the magnetocrystalline anisotropy *K*_1_ in Fe_1−*x*_Ni*_x_* alloys [data taken from the literature (Tarasov, 1939 ▸; Hall, 1960 ▸)]. Black dashed line: linear regression of *K*_1_(*x*). (*c*) Best-fit results for the residual scattering cross section dΣ_res_/dΩ (red diamonds), the scattering function *S*_H_ (orange open circles) and *S*_M_ (blue open circles). Blue solid line: fit of *S*_M_ assuming a Lorentzian-squared function for the *q*-dependence.

Fig. 6 ▸ shows the magnetic SANS results determined from the field-dependent approach described in Section 3.3. In the present case, to warrant the validity of the micromagnetic SANS theory, only dΣ/dΩ measured for μ_0_ *H*_i_ ≳ 65 mT (*i.e.* within the approach-to-saturation regime, compare Fig. 2 ▸) were considered. We have also restricted our neutron data analysis to 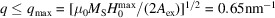 , since the magnetic SANS cross section is expected to be field-independent for *q* ≥ *q*_max_ (Michels, 2021 ▸). In Fig. 6 ▸(*a*), we plot the (over 2π) azimuthally averaged dΣ/dΩ along with the corresponding fits based on the micromagnetic SANS theory [Equation (10), black solid lines]. It is seen that the field dependence of dΣ/dΩ over the restricted *q*-range can be well reproduced by the theory. Fig. 6 ▸(*b*) displays the (weighted) mean-squared deviation between experiment and fit, χ^2^, determined according to Equation (13), as a function of the exchange-stiffness constant *A*_ex_. In this way, we find *A*_ex_ = (10 ± 1) pJ m^−1^ (see Table 1 ▸). The comparison with previous studies is discussed in the next paragraph for more clarity. Fig. 6 ▸(*c*) displays the best-fit results for dΣ_res_/dΩ, *S*_H_ and *S*_M_. Not surprisingly, the magnitude of dΣ_res_/dΩ (limit of dΣ/dΩ at infinite field) is smaller than the dΣ/dΩ at the largest fields [compare Fig. 6 ▸(*a*)], supporting the validity of the micromagnetic SANS theory. Furthermore, the magnitude of *S*_H_ is about two orders of magnitude smaller than *S*_M_, suggesting that the magnetization jump Δ*M* at internal particle–matrix interfaces represents the main source of spin disorder in this material. The estimated values for the mean-square anisotropy field and the mean-square magnetostatic field in terms of Equation (14) are 0.3 and 24 mT, respectively. These values qualitatively support the notion of dominant spin-misalignment scattering due to magnetostatic fluctuations. The *q*-dependence of *S*_M_ can be described using a Lorentzian-squared function [blue solid line in Fig. 6 ▸(*c*)] from which an estimate for the magnetostatic correlation length ξ_M_ = 2.4 ± 0.2 nm is obtained. This value compares favorably with the value of *l*_M_ = (2*A*_ex_/μ_0_ *M*_S_^2^)^1/2^ = 3.7 nm [using *A*_ex_ = 10 pJ m^−1^ and μ_0_ *M*_S_ = 1.34 T (taken from Table 1 ▸)], which reflects the competition between the exchange and magnetostatic energies.

We would like to emphasize that our experimental value for *A*_ex_ = 10 pJ m^−1^ is about 2–3 times larger than those reported in NANOPERM-type soft magnetic materials (Honecker *et al.*, 2013 ▸). Since the Curie temperature of the residual amorphous phase in our nanocrystalline (Fe_0.7_Ni_0.3_)_86_B_14_ sample is well above 700 K (see Fig. 3 ▸ and Table 1 ▸), while that of the Fe_89_Zr_7_B_3_Cu_1_ sample used in the previous study (Honecker *et al.*, 2013 ▸) was as low as 350 K, the local exchange stiffness in the grain boundary amorphous phase in HiB-NANOPERM-type alloys is expected to be higher than that in NANOPERM-type alloys. This finding could explain the origin of the larger *A*_ex_ value reported in the present study. Therefore, one can expect an improvement of the magnetic softness in HiB-NANOPERM thanks to the ensuing increase of the ferromagnetic exchange length *L*_0_. It is well established that nonmagnetic and/or ferromagnetic additives and the annealing conditions strongly affect the microstructural and magnetic properties of Fe-based nanocrystalline materials (McHenry *et al.*, 1999 ▸; Herzer, 2007 ▸, 2013 ▸; Suzuki *et al.*, 2019 ▸) and therefore have a strong impact on their magnetic softness. Using *A*_ex_ = 10 pJ m^−1^ (this study), *K*_1_ ≃ 9.0 kJ m^−3^,^1^ and φ_0_ ≃ 1.5 (Herzer, 2007 ▸), we obtain *L*_0_ ≃ 50 nm. This value for *L*_0_ is in very good agreement with the typical length scale of ∼30–50 nm previously reported in soft magnetic Fe-based alloys. Moreover, the comparison of the average grain size *D* = 7 nm with the *L*_0_ value, here *D* ≪ *L*_0_, also confirms that in the framework of the random anisotropy model (Herzer, 1989 ▸, 1990 ▸, 2007 ▸; Suzuki *et al.*, 1998 ▸), the exchange-averaged magnetic anisotropy 〈*K*〉 falls into the regime where 〈*K*〉 ∝ *D* ^3^. This finding is also consistent with the (experimental) *D* ^3^-dependence of *H*_C_ reported in Fe–B-based HiB-NANOPERM alloys (Suzuki *et al.*, 2019 ▸; Li *et al.*, 2020 ▸).

## 5. Conclusions

We employed magnetic SANS to determine the magnetic interaction parameters in (Fe_0.7_Ni_0.3_)_86_B_14_ alloy, which is a HiB-NANOPERM-type soft magnetic material. The analysis of the magnetic SANS data suggests the presence of strong spin misalignment on a mesoscopic length scale. In fact, the micromagnetic SANS theory provides an excellent description of the field dependence of the total (nuclear + magnetic) and purely magnetic SANS cross sections. The clover-leaf-type angular anisotropy patterns observed in the magnetic SANS signal can be well reproduced by the theory. The magnitudes of the scattering functions *S*_H_ and *S*_M_ allow us to conclude that the magnetization jumps at internal particle–matrix interfaces and the ensuing dipolar stray fields are the main source of the spin-disorder in this material. Our study highlights the strength of the magnetic SANS technique to characterize magnetic materials on the mesoscopic length scale. The structural and magnetic results (summarized in Table 1 ▸) provide valuable information on the (Fe_0.7_Ni_0.3_)_86_B_14_ ribbons, and further confirm the strong potential of Fe–Ni–B-based HiB-NANOPERM-type alloys as soft magnetic nanocrystalline materials. In the context of the random anisotropy model, we demonstrated that the magnetic softness in this system can be attributed to the combined action of the small particle size (*D* = 7 nm) and an increased exchange constant (*A*_ex_ = 10  pJ m^−1^) resulting in an enhanced exchange correlation length *L*_0_.

The data that support the findings of this study are available from the corresponding author upon reasonable request.

## Figures and Tables

**Table 1:** Summary of the structural and magnetic parameters for (Fe_0.7_Ni_0.3_)_86_B_14_ alloy (HiB-NANOPERM-type soft magnetic nanocrystalline material) determined by wide-angle XRD, magnetometry, DTA, TMGA and SANS

Parameter	(Fe_0.7_Ni_0.3_)_86_B_14_ alloy
*a* (nm)	∼0.359
*D* (nm)	7 ± 1
μ_0_ *M*_S_ (T)	1.34 ± 0.20
μ_0_ *H*_C_ (mT)	∼0.0049
T_{\rm C}^{\rm am} (K)	720
*A*_ex_ pJ m^−1^	10 ± 1
ξ_M_ (nm)	2.4 ± 0.2
*L*_0_ (nm)	∼50
μ_0_〈∣**H**_p_∣^2^〉^1/2^ (mT)	∼0.3
μ_0_〈∣*M*_*z*_∣^2^〉^1/2^ (mT)	∼24

## Footnotes

1 Estimated by assuming a linear regression of *K*_1_ in Fe_1−*x*_Ni*_x_* alloys for an Fe composition *x* between 0 and 0.4 at% [see inset in Fig. 6 ▸(*b*), data taken from the literature (Tarasov, 1939 ▸; Hall, 1960 ▸)].
